# Multi-Cell-Type Openness-Weighted Association Studies for Trait-Associated Genomic Segments Prioritization

**DOI:** 10.3390/genes13071220

**Published:** 2022-07-08

**Authors:** Shuang Song, Hongyi Sun, Jun S. Liu, Lin Hou

**Affiliations:** 1 Center for Statistical Science, Department of Industrial Engineering, Tsinghua University, Beijing 100084, China; song-s19@mails.tsinghua.edu.cn (S.S.); hy-sun18@mails.tsinghua.edu.cn (H.S.); 2 Department of Statistics, Harvard University, Cambridge, MA 02138, USA; 3 MOE Key Laboratory of Bioinformatics, School of Life Sciences, Tsinghua University, Beijing 100084, China

**Keywords:** multiple cell types, chromatin accessibility, aggregated Cauchy association test

## Abstract

Openness-weighted association study (OWAS) is a method that leverages the in silico prediction of chromatin accessibility to prioritize genome-wide association studies (GWAS) signals, and can provide novel insights into the roles of non-coding variants in complex diseases. A prerequisite to apply OWAS is to choose a trait-related cell type beforehand. However, for most complex traits, the trait-relevant cell types remain elusive. In addition, many complex traits involve multiple related cell types. To address these issues, we develop OWAS-joint, an efficient framework that aggregates predicted chromatin accessibility across multiple cell types, to prioritize disease-associated genomic segments. In simulation studies, we demonstrate that OWAS-joint achieves a greater statistical power compared to OWAS. Moreover, the heritability explained by OWAS-joint segments is higher than or comparable to OWAS segments. OWAS-joint segments also have high replication rates in independent replication cohorts. Applying the method to six complex human traits, we demonstrate the advantages of OWAS-joint over a single-cell-type OWAS approach. We highlight that OWAS-joint enhances the biological interpretation of disease mechanisms, especially for non-coding regions.

## 1. Introduction

Genome-wide association studies (GWAS) have been a powerful tool for identifying genetic signals associated with complex traits [1]. In recent years, numerous trait-related single nucleotide polymorphisms (SNPs) have been inferred from GWAS, which brings novel insights into disease mechanisms and genetic architectures. However, the majority of GWAS loci (∼89%) lie within non-coding regions [2,3]; hence, their interpretation remains a significant challenge. In recent years, methods incorporating functional annotations have been developed which support the prioritization of non-coding variants, improving the understanding of the biological mechanisms underlying complex traits [4,5].

Chromatin accessibility refers to the degree to which nuclear macromolecules are able to physically contact chromatinized DNA [6,7]. As an epigenetic feature, chromatin accessibility is an informative functional annotation in revealing active regulatory elements [8]. In addition, measurements of chromatin accessibility are predictive for gene expression [9,10]. However, obtaining experimental epigenome data for large cohorts is costly. As an alternative, computational approaches have been proposed for chromatin accessibility prediction. For example, deltaSVM leverages the gkm-SVM classifier and quantifies cell-type-specific effects of variants on DNase I sensitivity in their native genomic contexts [11]. DeepCage improves the prediction of novel chromatin accessible regions with a unified deep neural network, which integrates both sequence information and the binding status of transcription factors [12].

Recently, we have developed the openness-weighted association studies (OWAS) approach, a computational framework that leverages predicted chromatin accessibility for the prioritization of GWAS signals [13]. The first step in OWAS is to choose a trait-related cell type (e.g., the liver for low-density lipoprotein (LDL), and whole-blood for Crohn’s disease (CD)). However, selecting an optimal cell type for a given complex trait remains a substantial challenge [14,15]. In fact, many complex traits involve multiple related cell types, such as obesity being regulated by cells in both brain and adipose tissues [3,16,17]. Additionally, it has been shown that phenotype-genotype associations are enriched in open regions in multiple cell types for many traits [18,19]. Specifically, associations with height displayed relatively ubiquitous enrichments in DNase I hypersensitive sites of cell types including blood, fetal muscle, pancreas, etc. Even for those traits that showed relatively narrow enrichments, such as CD, the associations were also enriched in open regions of fibroblast and fetal intestine, in addition to blood cells [19]. Therefore, it is reasonable to incorporate information from multiple cell types to improve the performance of single-cell-type OWAS analysis.

In this study, we introduce OWAS-joint, a computational framework that integrates multi-cell-type chromatin accessibility predictions to prioritize trait-associated genomic segments. OWAS-joint aggregates single-cell-type OWAS test statistics via an aggregated Cauchy association test (ACAT) [20], bypassing the selection of specific cell types. We show through simulations that OWAS-joint improves statistical power compared to single-cell-type methods. We compared the performance of OWAS-joint and single-cell-type with real data applications for six complex traits, including CD, rheumatoid arthritis (RA), hypertension (HT), prostate cancer (PrCa), high-density lipoprotein (HDL), and LDL. Compared with OWAS, OWAS-joint identified more signals that were biologically interpretable. Furthermore, segments identified by OWAS-joint explain higher heritability and have high replication rates in independent cohorts. OWAS-joint takes GWAS summary statistics as the input and can be easily applied to large cohorts (e.g., UK Biobank, UKBB). The findings of OWAS-joint highlight the mediating effects of chromatin accessibility to the phenotype of interest, which improves the functional interpretation of non-coding genetic variants and provides novel insights into disease mechanisms. We provide an R package, OWASjoint, to implement the proposed method, which is available at https://github.com/shuangsong0110/OWASjoint (accessed on 1 May 2022).

## 2. Materials and Methods

### 2.1. Jointly Modeling Multi-Cell-Type Openness Scores

**Figure 1:**
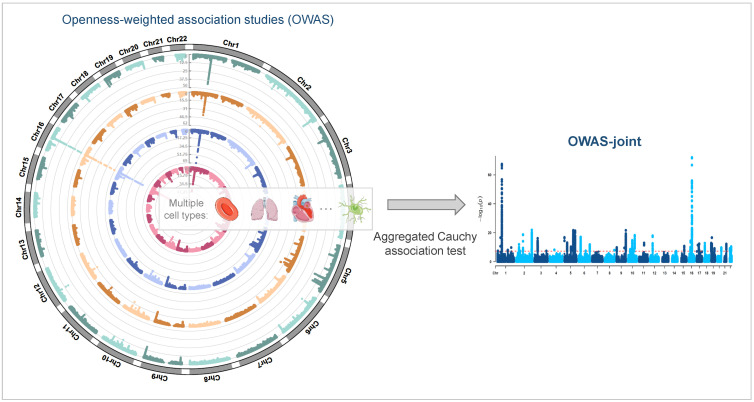
Schematic diagram of OWAS-joint. Cell-type-specific predictions of personalized chromosome accessibility and GWAS summary statistics are integrated via OWAS. OWAS-joint aggregates the OWAS results with single cell types via an aggregated Cauchy association test.

OWAS-joint integrates in silico predictions of chromatin accessibility across multiple cell types and GWAS summary statistics to prioritize trait-associated genomic segments (Figure 1). We examine 100 KB up and down-stream from the transcription start sites of genes as regulatory regions, which cover most of the regulatory variants [21]. The regulatory regions are then divided into segments of 5 KB, and the results are robust to the choice of length [13]. In the software, users can specify a candidate segment either by length or by the number of SNPs covered. For each segment and each cell type, OWAS test statistics are calculated as described in Song et al. (2021). OWAS-joint combines the single-cell-type OWAS results with ACAT [20]. To elaborate, denote $p_{l,s}$ as the OWAS *p*-value of segment *s* concerning the *l*-th cell type. Specifically, the ACAT test statistic for segment *s* is

(1) $$T_{s}=\sum_{l=1}^{L}\left\{\eta_{l}\tan\left[(0.5-p_{l,s})\pi \right]\right\},$$

where *L* is the total number of cell types to be aggregated, and the $\eta_{l}$ values are non-negative weights that sum to 1, modeling the relative importance of each cell type. In particular, OWAS-joint is equivalent to single-cell-type OWAS on the *k*-th cell type when the weight of the *k*-th cell type is 1 and the weights of other cell types are 0. We set $\eta_{l}=\frac{1}{L}$ (l=1,⋯,L) in the following context assuming that there is no prior knowledge on the related cell types. The transformed quantity $\tan\left[(0.5-p_{l,s})\pi \right]$ follows the Cauchy distribution if the *p*-value $p_{l,s}$ is from the null (i.e., $p_{l,s}$ follows a uniform distribution). Then the test statistic $T_{s}$, which is a weighted average of Cauchy random variables, has approximately a Cauchy tail under the null regardless of the dependency structure [20,22]. Thus, we can transform $T_{s}$ based on the cumulative density function of the Cauchy distribution to derive the *p*-value of the multi-cell-type OWAS test,

(2) $$p_{s}\approx \frac{1}{2}-\frac{\arctan(T_{s})}{\pi }.$$

We note that the approximation holds with arbitrary correlation structures of the OWAS test statistics across cell types, which ensures the computational efficiency of OWAS-joint.

The *p*-value cut-offs for segment-level association tests were determined using the Bonferroni correction. For each cell type, we used 0.05 divided by the total number of segments as the significance threshold (∼$5\times 10^{-8}$) to identify the trait-associated genomic segments.

### 2.2. Linkage Disequilibrium (LD) Shrinkage

In OWAS [13], the *z*-score for the *s*-th segment is derived by

(3) $$Z_{s}=\frac{{\hat{\gamma }}_{s}}{se({\hat{\gamma }}_{s})}\approx \sum_{j\in \Omega_{s}}w_{j}\frac{{\hat{\sigma }}_{j}}{{\hat{\sigma }}_{s}}z_{j},$$

where $\gamma_{s}$ is the effect size of segment *s* for the trait; $\Omega_{s}$ denotes the set of SNPs in segment *s*; $w_{j}$, $z_{j}$, and $\sigma_{j}^{2}$ denote the openness effect, *z*-score, and sample variance of the *j*-th SNP, respectively, and $\sigma_{s}^{2}$ is the sample variance of the openness scores in segment *s*. We estimate $\sigma_{s}^{2}$ with ${\hat{\sigma }}_{s}^{2}=W_{s}^{T}\hat{Cov}\left(X_{s}\right)W_{s}$, where $W_{s}$ is the vector containing the openness effects of SNPs in segment *s*. The covariance matrix of the genotype, $Cov(X_{s})$, can be estimated from an external LD reference panel, such as the 1000 Genomes Project (1000G) [23] and UKBB [24].

In real data applications, the LD matrix was estimated from European samples from the 1000G reference panel. However, the limited sample size leads to rank deficiency of the estimated covariance matrix. The singularity of the covariance matrix may yield a $\sigma_{s}^{2}$ close to zero, and further cause inflation of $Z_{s}$. Therefore, we leverage a two-step procedure to improve the robustness of the proposed method. In the first step, we perform an LD-based clumping using PLINK software with an LD threshold of 0.99 to exclude SNPs with perfect LD [25]. Secondly, we compute a shrinkage estimate of the LD matrix using the R package “corpcor” [26,27]. After shrinkage, the estimated covariance matrix is always positive definite and well-conditioned.

### 2.3. Simulation Settings

We used genotype data from the Wellcome Trust Case Control Consortium (WTCCC) dataset in simulation studies (*n* = 15,757) [28]. The predicted openness weights of three cell types, Th1, GM12878, and A549, were used as true openness effects in simulations to capture the genetic architecture of openness effects. For each segment, the openness score for the *l*-th cell type (l=1,2,3) is:

(4) $$O_{l}=XW_{l},$$

where X is the genotype matrix of the segment, and $W_{l}$ is the openness weights vector for SNPs in the segment of the *l*-th cell type. A quantitative phenotype is generated using the openness scores of the segment of all three cell types, i.e.,

(5) $$y=\lambda_{1}O_{1}+\lambda_{2}O_{2}+\lambda_{3}O_{3}+\epsilon ,$$

where $\lambda_{l}$ is the effect size of the openness score of the *l*-th cell type. The error terms ϵ are identical and are independently normally distributed.

To evaluate the type-I error rate, we set $\lambda_{1}=\lambda_{2}=\lambda_{3}=0$, which indicates the phenotype is not associated with any openness scores. With respect to the evaluation of statistical power, we varied the simulated genetic architectures in two respects: the number of causal cell types, and the heritability explained by the segment. Specifically, the number of causal cell types was decided by adjusting $\lambda_{l}$ with

1. $\lambda_{1}=1,\lambda_{2}=\lambda_{3}=0.$ Only the first cell type (Th1) is causal to the phenotype.
2. $\lambda_{1}=\lambda_{2}=\frac{1}{2},\lambda_{3}=0$. The cell types Th1 and GM12878 are causal, while A549 is not.
3. $\lambda_{1}=\lambda_{2}=\lambda_{3}=\frac{1}{3}$. All three cell types are causal.

The heritability was set to 0.02% for the low heritability setting, and 0.1% for the high heritability effect setting. We removed segments containing fewer than two SNPs in simulations. The procedure was repeated 10 times on 500 randomly selected segments on chromosome 1.

### 2.4. GWAS Datasets

#### 2.4.1. GWAS Summary Statistics

We provide the details of GWAS data used in our analysis in Table S1. The UKBB GWAS summary statistics were downloaded from the second round of results released in August 2018 by the Neale group (http://www.nealelab.is/uk-biobank, accessed on 3 February 2021). The Genetic Epidemiology Research on Aging (GERA) summary statistics were used for replication analysis for HT (dbGaP: phs000674.v3.p3, http://cg.bsc.es/gera_summary_stats/, accessed on 9 October 2021).

#### 2.4.2. Individual-Level Genotype Data

We used individual-level genotype data from WTCCC for simulations and real data applications to compute heritability. For quality control, we removed SNPs with a genotyping failure rate larger than 0.02 and significant Hardy–Weinberg equilibrium with $p<10^{-6}$ in PLINK 1.9 [25]. We also removed samples with a missing rate larger than 0.02. We excluded the HLA region (chr6: 28,477,797-33,448,354, hg19) in the heritability estimation.

### 2.5. Predicted Openness of Personal Genomes

We obtained the predicted openness effect ($w_{j}$) of each SNP via the deltaSVM [11]. The model was trained on DNase I-hypersensitive sites of 12 cell types from the UW ENCODE Project (http://www.beerlab.org/deltasvm/, accessed on 13 May 2021). A list of the cell types is provided in Table S2.

### 2.6. Pathway Enrichment Analysis

We tested the enrichment of OWAS genes in KEGG pathways [29] using the R package “clusterProfiler” [30]. The false discovery rate (FDR) was controlled at the level of 0.2 with an R package “*q*-value” [31,32].

## 3. Results

### 3.1. Simulations

**Figure 2:**
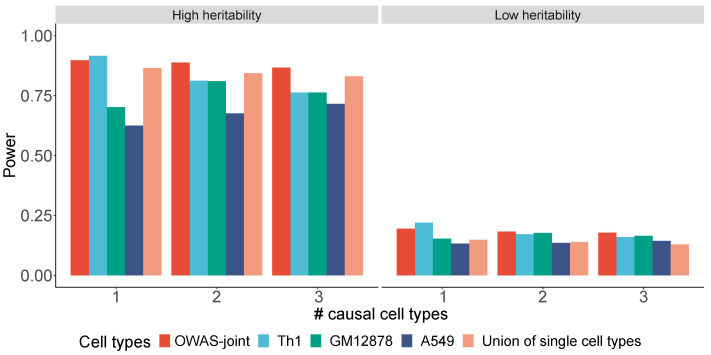
The statistical power of OWAS-joint, OWAS applied with each of the three cell types, and a union of single-cell-type methods with Bonferroni correction. Both phenotype effects with high (0.1%) and low (0.02%) heritability were considered. The simulation settings 1, 2, and 3 correspond to one (Th1), two (Th1 and GM12878), and three causal cell types.

A key improvement of the OWAS-joint framework is to aggregate the statistical evidence across multiple cell types. We performed simulations to evaluate the type-I error rate and statistical power of OWAS-joint under varying genetic settings. The openness weights from three commonly used cell types, A549, GM12878, and Th1, were considered in the simulations (Section 2). In the null simulations, type-I error rates for both OWAS-joint and single-cell-type OWAS were well controlled (Table S3). Under various heritability settings, the statistical power was improved by OWAS-joint when the phenotype was correlated with the openness scores of multiple cell types (Figure 2, settings 2–3). Even when only one cell type was causal, the statistical power of OWAS-joint was comparable to the results based on the causal cell type (Figure 2, setting 1). We also compared the statistical power of OWAS-joint to simply combining results across three cell types with the Bonferroni correction. OWAS-joint achieved a consistent power improvement from 30.9% to 38.0% under low heritability settings, and 3.7% to 5.2% under high heritability settings.

### 3.2. Real Data Applications

#### 3.2.1. OWAS-Joint Identifies More Genetic Signals

For real data applications, we applied OWAS-joint to six complex traits including CD [33], RA [34], HT [24], PrCa [35], HDL [36], and LDL [36]. A detailed description of the GWAS datasets is provided in Table S1. We compared the results of OWAS-joint to that of single-cell-type OWAS, based on the 12 common cell types from the UW ENCODE Project (Section 2). Consistent with the simulation studies, OWAS-joint identified more trait-associated genes and segments than the single-cell-type OWAS and the union with Bonferroni correction (Table 1 and Figure S1). For example, OWAS-joint identified 382 significant genes for CD, while the single-cell-type OWAS identified 293 genes on average (standard deviation, SD =13). The same patterns were observed in the analysis of segments, where OWAS-joint identified 2743 trait-associated segments. In contrast, on average, the single-cell-type OWAS identified 1138 segments (SD = 48).

#### 3.2.2. OWAS-Joint Provides Novel Biological Interpretation

We first performed KEGG pathway enrichment analysis on genes identified by OWAS-joint and single-cell-type OWAS analysis (Figures S2 and S3). The related cell type for single-cell-type OWAS was pre-specified based on domain knowledge [13,19,37] (Table S1). Here, we focus our discussions on pathways that were enriched in OWAS-joint genes but not in single-cell-type OWAS genes. The two infection-related pathways, the hepatitis B (*q*-value = 0.14) and measles (*q*-value = 0.16) pathways, were enriched for CD. Infectious agents were implicated in the initiation, maintenance, and risk of chronic inflammation in CD [38,39]. The antigen processing and presentation pathway (*q*-value = 0.16) was also enriched in OWAS-joint genes for CD, which confirms the pivotal role of antigen-presenting cells in intestinal inflammation [40]. Another example is the PPAR signaling pathway, which was enriched in OWAS-joint genes for LDL (*q*-value = 0.12). As discussed in Gusev et al. (2018), *PPARG* activation increases LDL metabolism via induction of *LDLR* and *CYP7A1* [41]. It has also been reported that *PPAR* agonists decrease glycated LDL uptake into macrophages via regulation of lipoprotein lipase [42]. We also found the prostate cancer pathway was enriched in OWAS-joint genes for PrCa (*q*-value = 0.17), which validates the results.

We further show that OWAS-joint identifies more candidate genes than single-cell-type OWAS. We used 17 candidate causal genes-phenotype pairs for CD and HDL in Wainberg et al. (2019) to benchmark OWAS-joint and OWAS. For CD, OWAS-joint identified all six candidate causal genes, whereas *STAT3* (OWAS-joint *p*-value = $2.3\times 10^{-8}$) was not identified by the single-cell-type OWAS with the whole-blood cell type (Th1). Experiments have shown that *STAT3*-knockout mice develop Crohn’s-like symptoms [43]. For LDL, OWAS-joint identified 8 out of 11 candidate causal genes, while OWAS with liver cell type (HepG2) only identified 7 with *PPARG* missed (OWAS-joint *p*-value = $8.3\times 10^{-9}$).

OWAS-joint genes can also be validated with etiological evidence. We highlight several examples of CD. As discussed in Verlaan et al. (2009), the allele-specific chromatin remodeling in *ZPBP2* (OWAS-joint *p*-value = $2.1\times 10^{-10}$) is associated with the risk of autoimmune disease [44]. Furthermore, *LRRK2* (OWAS-joint *p*-value = $9.0\times 10^{-16}$) was found to suppress the transcriptional activity of *NFAT1*, which has been considered a key target for treating immune disorders [45]. In addition, we also found some susceptible genes for ulcerative colitis (UC). UC is known to have a high genetic correlation (around 0.7) with CD [46]. An example is *GNAI2* ( OWAS-joint *p*-value = $2.5\times 10^{-9}$), which plays an essential role in the inflammatory process [47]. *GNAI2*-deficient mice develop a lethal diffuse colitis with clinical and histopathological features, which is similar to UC in humans [48].

#### 3.2.3. More Heritability Explained by OWAS-Joint Segments

**Figure 3:**
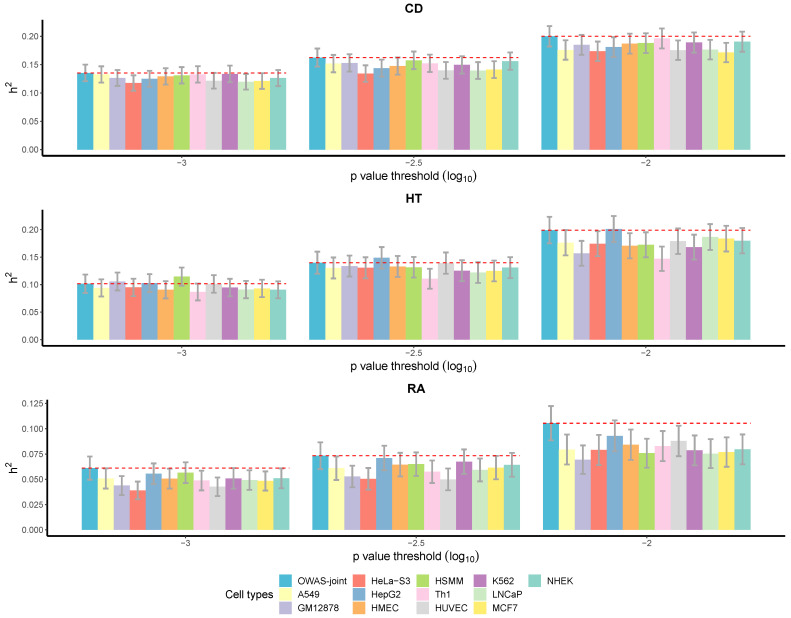
Heritability ($h^{2}$) explained by OWAS-joint segments and single-cell-type OWAS segments. Varying *p*-value thresholds were considered. The error bars correspond to the standard error of the heritability estimated by GCTA software. The heritability was evaluated with the WTCCC individual-level genotype data. The red dashed lines mark the heritability explained by OWAS-joint segments.

As discussed in Song et al. (2021), OWAS segments explain more heritability compared with the genes or annotated SNP sets found by other methods, such as FUSION [49] and CADD [50]. In the following, we show that OWAS-joint segments explain more heritability than single-cell-type OWAS. Heritability was evaluated with WTCCC individual-level genotype data for CD, HT, and RA. The samples for evaluation were independent of the samples in the discovery cohorts. From Figure 3, we can see that the heritability explained by OWAS-joint segments was higher or comparable to the highest heritability explained by the single-cell-type OWAS segments with each of the 12 cell types. The patterns were consistent across the three traits under varying *p*-value thresholds, and the improvement was especially notable for RA.

#### 3.2.4. Replication Rates of OWAS-Joint Segments

**Figure 4:**
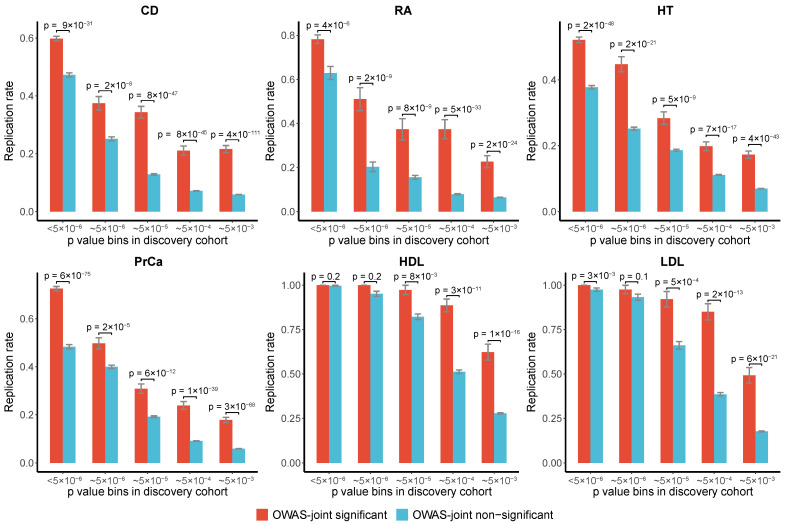
Replication rates of OWAS-joint results. OWAS-joint was performed with GWAS summary statistics on CD, RA, HT, PrCa, HD, and LDL from the discovery cohort (with larger sample sizes) and the replication cohort from UKBB and GERA. In the discovery cohort, GWAS SNPs were divided into five bins according to their *p*-values (I: ($0.5\times 10^{-6}$), II: [$5\times 10^{-6}$, $5\times 10^{-5}$), III: [$5\times 10^{-5}$, $5\times 10^{-4}$), IV: [$5\times 10^{-4}$, $5\times 10^{-3}$), V: [$5\times 10^{-3}$, 0.05)). In the replication cohort, GWAS significant SNPs were identified with a relaxed threshold (*p*<0.05). In each bin, SNPs were broken down into prioritized and non-prioritized groups by the OWAS-joint results ($p<5\times 10^{-8}$). The *p*-values shown in the figure were derived from the binomial test.

We evaluated the replication rates of OWAS-joint segments of the six traits mentioned above. The independent GWAS cohort details for evaluating replication rates are provided in Table S4. We first binned the SNPs by GWAS *p*-values in the discovery cohorts. For SNPs, either identified by OWAS-joint (i.e., SNPs that are harbored by significant segments identified by OWAS-joint) or not, we calculated the replication rate in the replication GWAS cohort within each bin. OWAS-joint achieved high replication rates for all six phenotypes and all *p*-value bins (Figure 4). This indicated that OWAS-joint segments effectively detected the truly associated SNPs. The improvement in the replication rates was more prominent for GWAS SNPs with moderate *p*-values, which indicated the benefits of aggregating multiple cell types when the GWAS power is limited.

## 4. Discussion

Despite the success of GWAS in identifying tens of thousands of associations between SNPs and complex traits, the interpretation of GWAS signals remains challenging. The growing amount of cell-type-level epigenomic data has increased our understanding of non-coding variations, but it is still unclear which cell types are more important for the development of complex traits. In this article, we propose an efficient statistical framework, aggregating personalized chromatin accessibility across multiple cell types, for prioritization of disease-related genomic segments. OWAS-joint bypasses the selection of trait-related cell types, which addresses the main challenge of cell type selection in OWAS analysis. OWAS-joint tests the mediating effects of chromatin accessibility by quantifying the association between personalized openness across multiple cell types and the phenotype of interest. The results of OWAS-joint provide a biological interpretation, especially for non-coding variants, and promote our understanding of disease mechanisms.

In both simulations and real data applications, we demonstrated that OWAS-joint improves the statistical power and identifies more potential genetic signals than single-cell-type OWAS analysis. In addition, OWAS-joint segments were shown to have greater heritability enrichment and replication rates than OWAS. OWAS-joint takes GWAS summary statistics as input, which guarantees its general applicability without privacy concerns. The method can be easily applied to large cohorts (e.g., UKBB), and maintains high computational efficiency. The computational framework can also be extended to other epigenetic features for understanding of the mediation effects of epigenetic features for complex traits.

There are several limitations of our method. First, similar to OWAS, OWAS-joint is based on the openness effects predicted by machine learning methods. Therefore, its performance is affected by the prediction accuracy of the openness effects. When provided with more data sources (e.g., RNA-seq or Chip-seq data [51,52]), it is expected that the openness will be predicted more accurately. A recent study has shown that integrating sequence information and the binding status of transcription factors further improved the prediction accuracy of chromatin accessibility [12]. Second, we assume an additive model for the openness effect at SNP level. It may be of value to consider and evaluate the performance of other statistical models, such as dominant or recessive models. Third, our method provides a novel perspective to interpret non-coding variants by incorporating the change in chromatin accessibility in multiple cell types, but the method alone cannot infer causality. Fourth, we also note that the ACAT weights in Equation (1) can be flexibly specified based on domain knowledge of the relativity between the cell type and the trait. Future work is needed to design optimal weights based on data and background knowledge to further improve the performance of OWAS-joint.

## Figures and Tables

**Table 1:** The number of segments and genes identified by OWAS-joint, the union of single-cell-type OWAS with 12 cell types, and the average number of single-cell-type OWAS. The standard deviations across 12 cell types are shown in brackets. The *p*-value cutoffs for the union of segment-level association tests were determined by Bonferroni correction. The largest numbers of identified signals are highlighted in boldface.

Trait	# Identified Segments			# Identified Genes		
	OWAS-Joint	Union (Bonferroni)	Single-Cell-Type OWAS	OWAS-Joint	Union (Bonferroni)	Single-Cell-Type OWAS
CD	**2743**	2595	1138 (48)	**382**	374	293 (13)
RA	**1571**	1558	659 (54)	**595**	590	204 (16)
HT	**6598**	6308	2452 (111)	**978**	944	776 (18)
PrCa	**1711**	1650	635 (32)	**301**	293	213 (14)
HDL	**3070**	2944	1347 (41)	**1293**	1264	441 (23)
LDL	**2811**	2734	1196 (50)	**1229**	1219	399 (22)

## Data Availability

Publicly available datasets were analyzed in this study. The GWAS summary statistics data were downloaded from IBDG Consortium [33] (accessed on 30 August 2018); Stahl et al. (2010) [34] (accessed on 30 August 2018); Teslovich et al. [36] (accessed on 8 September 2021); Schumacher et al. (2018) [35] (accessed on 8 September 2021). The UKBB GWAS summary statistics were downloaded from the second round of results released in August 2018 by the Neale group (http://www.nealelab.is/uk-biobank, accessed on 3 February 2021). The GERA summary statistics were downloaded from http://cg.bsc.es/gera_summary_stats/ (dbGaP accession number: phs000674.v3.p3, accessed on 9 October 2021). The openness model was trained on DNase I-hypersensitive sites of 12 cell types from the UW ENCODE with deltaSVM Project (http://www.beerlab.org/deltasvm/, accessed on 13 May 2021). The individual-level genotype data from WTCCC are available at www.wtccc.org.uk (accessed on 1 Match 2020).

## Supplementary Materials

The following are available online at https://www.mdpi.com/article/10.3390/genes13071220/s1. Figure S1: The number of significant genes identified by OWAS-joint and OWAS with each of the 12 common cell types. Figure S2: KEGG pathway enrichment analysis results of OWAS-joint genes. Figure S3: KEGG pathway enrichment analysis results of single-cell-type OWAS genes. Table S1: GWAS summary statistics used in OWAS-joint and single-cell-type OWAS analysis. Table S2: Summary information of the 12 common human cell types from UW ENCODE and the corresponding tissues in OWAS analysis in simulations. Table S3: Type-I error rates of OWAS-joint, single-cell-type OWAS with each of the three cell types, and a union of single-cell-type methods with Bonferroni correction. Table S4: GWAS summary statistics for replication studies.

## Abbreviations

The following abbreviations are used in this manuscript:

1000G	1000 Genomes Project
ACAT	Aggregated Cauchy association test
CD	Crohn’s disease
GWAS	Genome wide association studies
HDL	High-density lipoprotein
HT	Hypertension
LD	Linkage disequilibrium
LDL	Low-density lipoprotein
OWAS	Openness-weighted association studies
PrCa	Prostate cancer
RA	Rheumatoid arthritis
SD	Standard deviation
SNP	Single nucleotide polymorphism
UKBB	UK Biobank
UC	Ulcerative colitis
WTCCC	Wellcome Trust Case Control Consortium

## References

[1] Jostins L., Barrett J.C. (2011). Genetic risk prediction in complex disease. Hum. Mol. Genet..

[2] Gusev A., Lee S.H., Trynka G., Finucane H., Vilhjálmsson B.J., Xu H., Zang C., Ripke S., Bulik-Sullivan B., Stahl E. (2014). Partitioning heritability of regulatory and cell-type-specific variants across 11 common diseases. Am. J. Hum. Genet..

[3] Cano-Gamez E., Trynka G. (2020). From GWAS to function: Using functional genomics to identify the mechanisms underlying complex diseases. Front. Genet..

[4] Hou L., Zhao H. (2013). A review of post-GWAS prioritization approaches. Front. Genet..

[5] Gallagher M.D., Chen-Plotkin A.S. (2018). The post-GWAS era: From association to function. Am. J. Hum. Genet..

[6] Klemm S.L., Shipony Z., Greenleaf W.J. (2019). Chromatin accessibility and the regulatory epigenome. Nat. Rev. Genet..

[7] Minnoye L., Marinov G.K., Krausgruber T., Pan L., Marand A.P., Secchia S., Greenleaf W.J., Furlong E.E., Zhao K., Schmitz R.J. (2021). Chromatin accessibility profiling methods. Nat. Rev. Methods Prim..

[8] Henikoff S., Henikoff J.G., Kaya-Okur H.S., Ahmad K. (2020). Efficient chromatin accessibility mapping in situ by nucleosome-tethered tagmentation. eLife.

[9] Pique-Regi R., Degner J.F., Pai A.A., Gaffney D.J., Gilad Y., Pritchard J.K. (2011). Accurate inference of transcription factor binding from DNA sequence and chromatin accessibility data. Genome Res..

[10] Ramachandran P., Palidwor G.A., Perkins T.J. (2015). BIDCHIPS: Bias decomposition and removal from ChIP-seq data clarifies true binding signal and its functional correlates. Epigenet. Chromatin.

[11] Lee D., Gorkin D.U., Baker M., Strober B.J., Asoni A.L., McCallion A.S., Beer M.A. (2015). A method to predict the impact of regulatory variants from DNA sequence. Nat. Genet..

[12] Liu Q., Hua K., Zhang X., Wong W.H., Jiang R. (2022). DeepCAGE: Incorporating transcription factors in genome-wide prediction of chromatin accessibility. Genom. Proteom. Bioinform..

[13] Song S., Shan N., Wang G., Yan X., Liu J.S., Hou L. (2021). Openness weighted association studies: Leveraging personal genome information to prioritize non-coding variants. Bioinformatics.

[14] Calderon D., Bhaskar A., Knowles D.A., Golan D., Raj T., Fu A.Q., Pritchard J.K. (2017). Inferring relevant cell types for complex traits by using single-cell gene expression. Am. J. Hum. Genet..

[15] Jiang L., Xue C., Dai S., Chen S., Chen P., Sham P.C., Wang H., Li M. (2019). DESE: Estimating driver tissues by selective expression of genes associated with complex diseases or traits. Genome Biol..

[16] Boyle E.A., Li Y.I., Pritchard J.K. (2017). An expanded view of complex traits: From polygenic to omnigenic. Cell.

[17] Schaid D.J., Chen W., Larson N.B. (2018). From genome-wide associations to candidate causal variants by statistical fine-mapping. Nat. Rev. Genet..

[18] Kundaje A., Meuleman W., Ernst J., Bilenky M., Yen A., Heravi-Moussavi A., Kheradpour P., Zhang Z., Wang J., Ziller M.J. (2015). Integrative analysis of 111 reference human epigenomes. Nature.

[19] Iotchkova V., Ritchie G.R., Geihs M., Morganella S., Min J.L., Walter K., Timpson N.J., Dunham I., Birney E., Soranzo N. (2019). GARFIELD classifies disease-relevant genomic features through integration of functional annotations with association signals. Nat. Genet..

[20] Liu Y., Chen S., Li Z., Morrison A.C., Boerwinkle E., Lin X. (2019). ACAT: A fast and powerful p value combination method for rare-variant analysis in sequencing studies. Am. J. Hum. Genet..

[21] Nasser J., Bergman D.T., Fulco C.P., Guckelberger P., Doughty B.R., Patwardhan T.A., Jones T.R., Nguyen T.H., Ulirsch J.C., Lekschas F. (2021). Genome-wide enhancer maps link risk variants to disease genes. Nature.

[22] Liu Y., Xie J. (2020). Cauchy combination test: A powerful test with analytic p-value calculation under arbitrary dependency structures. J. Am. Stat. Assoc..

[23] The 1000 Genomes Project Consortium (2015). A global reference for human genetic variation. Nature.

[24] Sudlow C., Gallacher J., Allen N., Beral V., Burton P., Danesh J., Downey P., Elliott P., Green J., Landray M. (2015). UK biobank: An open access resource for identifying the causes of a wide range of complex diseases of middle and old age. PLoS Med..

[25] Chang C.C., Chow C.C., Tellier L.C., Vattikuti S., Purcell S.M., Lee J.J. (2015). Second-generation PLINK: Rising to the challenge of larger and richer datasets. Gigascience.

[26] Schäfer J., Strimmer K. (2005). A Shrinkage Approach to Large-Scale Covariance Matrix Estimation and Implications for Functional Genomics. Stat. Appl. Genet. Mol. Biol..

[27] Opgen-Rhein R., Strimmer K. (2007). Accurate ranking of differentially expressed genes by a distribution-free shrinkage approach. Stat. Appl. Genet. Mol. Biol..

[28] The Wellcome Trust Case Control Consortium (2007). Genome-wide association study of 14,000 cases of seven common diseases and 3000 shared controls. Nature.

[29] Kanehisa M., Araki M., Goto S., Hattori M., Hirakawa M., Itoh M., Katayama T., Kawashima S., Okuda S., Tokimatsu T. (2007). KEGG for linking genomes to life and the environment. Nucleic Acids Res..

[30] Yu G., Wang L.G., Han Y., He Q.Y. (2012). clusterProfiler: An R package for comparing biological themes among gene clusters. Omics A J. Integr. Biol..

[31] Storey J.D., Tibshirani R. (2003). Statistical significance for genome-wide experiments. Proc. Natl. Acad. Sci. USA.

[32] Storey J.D., Xiao W., Leek J.T., Tompkins R.G., Davis R.W. (2005). Significance analysis of time course microarray experiments. Proc. Natl. Acad. Sci. USA.

[33] Liu J.Z., Van Sommeren S., Huang H., Ng S.C., Alberts R., Takahashi A., Ripke S., Lee J.C., Jostins L., Shah T. (2015). Association analyses identify 38 susceptibility loci for inflammatory bowel disease and highlight shared genetic risk across populations. Nat. Genet..

[34] Stahl E.A., Raychaudhuri S., Remmers E.F., Xie G., Eyre S., Thomson B.P., Li Y., Kurreeman F.A., Zhernakova A., Hinks A. (2010). Genome-wide association study meta-analysis identifies seven new rheumatoid arthritis risk loci. Nat. Genet..

[35] Schumacher F.R., Al Olama A.A., Berndt S.I., Benlloch S., Ahmed M., Saunders E.J., Dadaev T., Leongamornlert D., Anokian E., Cieza-Borrella C. (2018). Association analyses of more than 140,000 men identify 63 new prostate cancer susceptibility loci. Nat. Genet..

[36] Teslovich T.M., Musunuru K., Smith A.V., Edmondson A.C., Stylianou I.M., Koseki M., Pirruccello J.P., Ripatti S., Chasman D.I., Willer C.J. (2010). Biological, clinical and population relevance of 95 loci for blood lipids. Nature.

[37] Wainberg M., Sinnott-Armstrong N., Mancuso N., Barbeira A.N., Knowles D.A., Golan D., Ermel R., Ruusalepp A., Quertermous T., Hao K. (2019). Opportunities and challenges for transcriptome-wide association studies. Nat. Genet..

[38] Sartor R.B. (2008). Microbial influences in inflammatory bowel diseases. Gastroenterology.

[39] Spiller R., Campbell E. (2006). Post-infectious irritable bowel syndrome. Curr. Opin. Gastroenterol..

[40] Stagg A., Hart A., Knight S., Kamm M. (2003). The dendritic cell: Its role in intestinal inflammation and relationship with gut bacteria. Gut.

[41] Gusev A., Mancuso N., Won H., Kousi M., Finucane H.K., Reshef Y., Song L., Safi A., McCarroll S., Neale B.M. (2018). Transcriptome-wide association study of schizophrenia and chromatin activity yields mechanistic disease insights. Nat. Genet..

[42] Sekar A., Bialas A.R., De Rivera H., Davis A., Hammond T.R., Kamitaki N., Tooley K., Presumey J., Baum M., Van Doren V. (2016). Schizophrenia risk from complex variation of complement component 4. Nature.

[43] Regev A., Teichmann S.A., Lander E.S., Amit I., Benoist C., Birney E., Bodenmiller B., Campbell P., Carninci P., Clatworthy M. (2017). Science forum: The human cell atlas. eLife.

[44] Verlaan D.J., Berlivet S., Hunninghake G.M., Madore A.M., Larivière M., Moussette S., Grundberg E., Kwan T., Ouimet M., Ge B. (2009). Allele-specific chromatin remodeling in the ZPBP2/GSDMB/ORMDL3 locus associated with the risk of asthma and autoimmune disease. Am. J. Hum. Genet..

[45] Chae C.S., Kim G.C., Park E.S., Lee C.G., Verma R., Cho H.L., Jun C.D., Yoo Y.J., Im S.H. (2017). NFAT1 regulates systemic autoimmunity through the modulation of a dendritic cell property. J. Immunol..

[46] Yang Y., Musco H., Simpson-Yap S., Zhu Z., Wang Y., Lin X., Zhang J., Taylor B., Gratten J., Zhou Y. (2021). Investigating the shared genetic architecture between multiple sclerosis and inflammatory bowel diseases. Nat. Commun..

[47] Zhang W.J., Koltun W.A., Tilberg A.F., Page M.J., Chorney M.J. (2000). Absence of GNAI2 codon 179 oncogene mutations in inflammatory bowel disease. Inflamm. Bowel Dis..

[48] Rudolph U., Finegold M.J., Rich S.S., Harriman G.R., Srinivasan Y., Brabet P., Boulay G., Bradley A., Birnbaumer L. (1995). Ulcerative colitis and adenocarcinoma of the colon in G*α*i2-deficient mice. Nat. Genet..

[49] Gusev A., Ko A., Shi H., Bhatia G., Chung W., Penninx B.W., Jansen R., De Geus E.J., Boomsma D.I., Wright F.A. (2016). Integrative approaches for large-scale transcriptome-wide association studies. Nat. Genet..

[50] Rentzsch P., Witten D., Cooper G.M., Shendure J., Kircher M. (2019). CADD: Predicting the deleteriousness of variants throughout the human genome. Nucleic Acids Res..

[51] The ENCODE Project Consortium (2012). An integrated encyclopedia of DNA elements in the human genome. Nature.

[52] Mundade R., Ozer H.G., Wei H., Prabhu L., Lu T. (2014). Role of ChIP-seq in the discovery of transcription factor binding sites, differential gene regulation mechanism, epigenetic marks and beyond. Cell Cycle.

[53] Yin L., Zhang H., Tang Z., Xu J., Yin D., Zhang Z., Yuan X., Zhu M., Zhao S., Li X. (2021). rMVP: A memory-efficient, visualization-enhanced, and parallel-accelerated tool for genome-wide association study. Genom. Proteom. Bioinform..

