# Supplementary material for: Multi-Cell-Type Openness-Weighted Association Studies for Trait-Associated Genomic Segments Prioritization
Source: Genes (Basel). 2022 Jul 8;13(7):1220. doi: 10.3390/genes13071220 (PMC9323627; doi:10.3390/genes13071220)
Supplement: Supplementary file 1 [file genes-13-01220-s001.zip › genes-1749182-supplementary.pdf]

Supplement to "Multi-cell-type openness-weighted association studies for trait-associated genomic segments prioritization"

Shuang Song, Hongyi Sun, Jun S. Liu, Lin Hou

Contents

1 Supplementary Tables

2 Supplementary Figures

1 Supplementary Tables

Table S1: GWAS summary statistics used in OWAS-joint and single-cell-type OWAS analysis. For each trait, a related cell type was selected by background knowledge for single-cell-type OWAS analysis.

Trait	Data	N_{case}	$N_{control}$	Related cell type
Crohn's disease (CD)	IBDG	20,550	41,642	Th1
Rheumatoid arthritis (RA)	Stahl et al. (2010)	5,539	20,169	GM12878
Hypertension (HT)	UK Biobank	93,560	267,581	Huvec
Prostate cancer (PrCa)	Schumacher et al. (2018)	46,939	27,910	Huvec
High-density lipoprotein (HDL)	Teslovich et al. (2010)	99,900		HepG2
Low-density lipoprotein (LDL)	Teslovich et al. (2010)	95,454		HepG2

Table S2: Summary information of the 12 common human cell types from UW ENCODE and the corresponding tissues in OWAS analysis in simulations.

Cell type	Tissue	Description
A549	Epithelium	Human lung carcinoma derived epithelial cell line
GM12878	Blood	Lymphoblastoid
HeLa-S3	Cervix	Adherent human cervical adenocarcinoma
HepG2	Liver	Hepatocellular carcinoma
HMEC	Breast	Mammary epithelial
HSMM	Muscle	Human skeletal muscle myoblasts
HUVEC	Blood vessel	Umbilical vein endothelial
K562	Blood	Myeloid
LNCaP	Prostate	Prostate adenocarcinoma
MCF-7	Breast	Mammary gland adenocarcinoma
NHEK	Skin	Normal epidermal keratinocytes
Th1	Blood	T1 helper

Table S3: Type-I error rates of OWAS-joint, single-cell-type OWAS with each of the three cell types, and a union of single-cell-type methods with Bonferroni correction.

α	Joint	Th1	GM12878	A549	Union of single cell types
0.010	0.009	0.009	0.008	0.008	0.006
0.020	0.019	0.019	0.017	0.018	0.011
0.030	0.031	0.031	0.029	0.030	0.015
0.040	0.041	0.040	0.039	0.039	0.022
0.050	0.050	0.051	0.048	0.049	0.028

Table S4: GWAS summary statistics for replication studies. The overlapped samples between the discovery cohorts and replication cohorts were removed.

Trait	Data	N_{case}	$N_{control}$
Crohn's disease (CD)	UK Biobank	1,096	360,045
Rheumatoid arthritis (RA)	UK Biobank	4,017	357,124
Hypertension (HT)	Genetic Epidemiology Research on Aging	28,391	28,246
Prostate cancer (PrCa)	UK Biobank	2,653	164,335
High-density lipoprotein (HDL)	UK Biobank	315,133	
Low-density lipoprotein (LDL)	UK Biobank	343,621	

2 Supplementary Figures

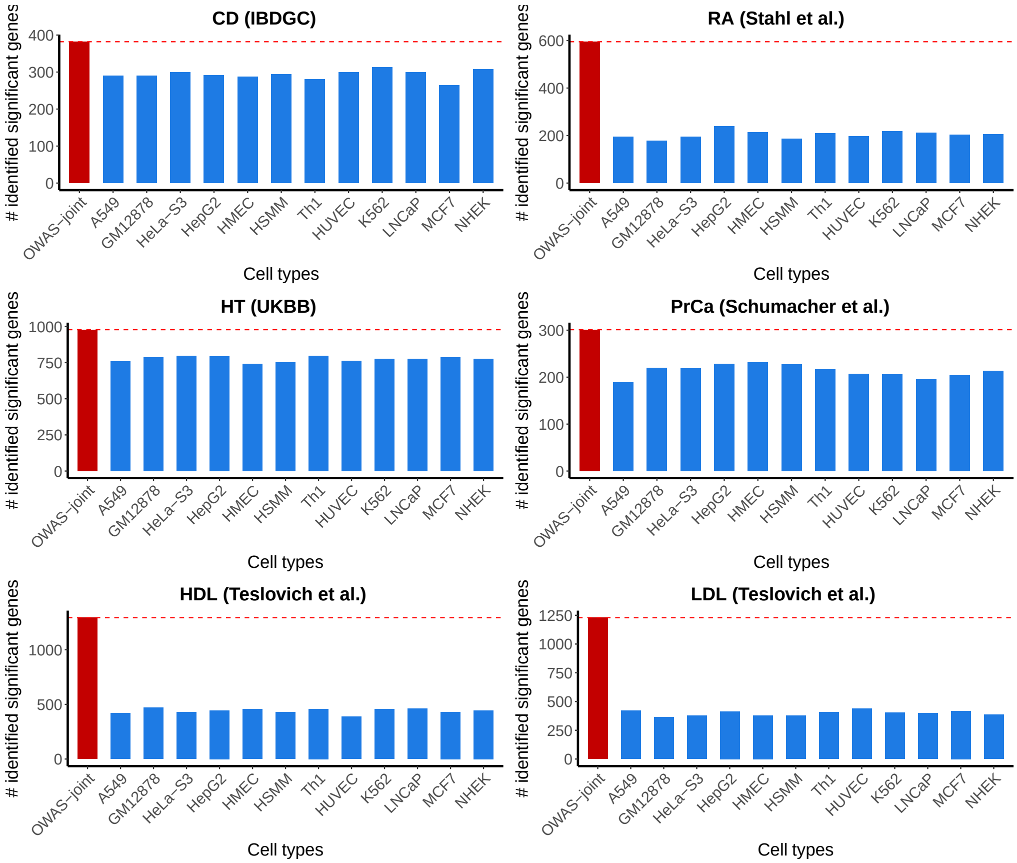

Figure S1: The number of significant genes identified by OWAS-joint and OWAS with each of the 12 common cell types. The red dashed line represents the number of genes identified by OWAS-joint.

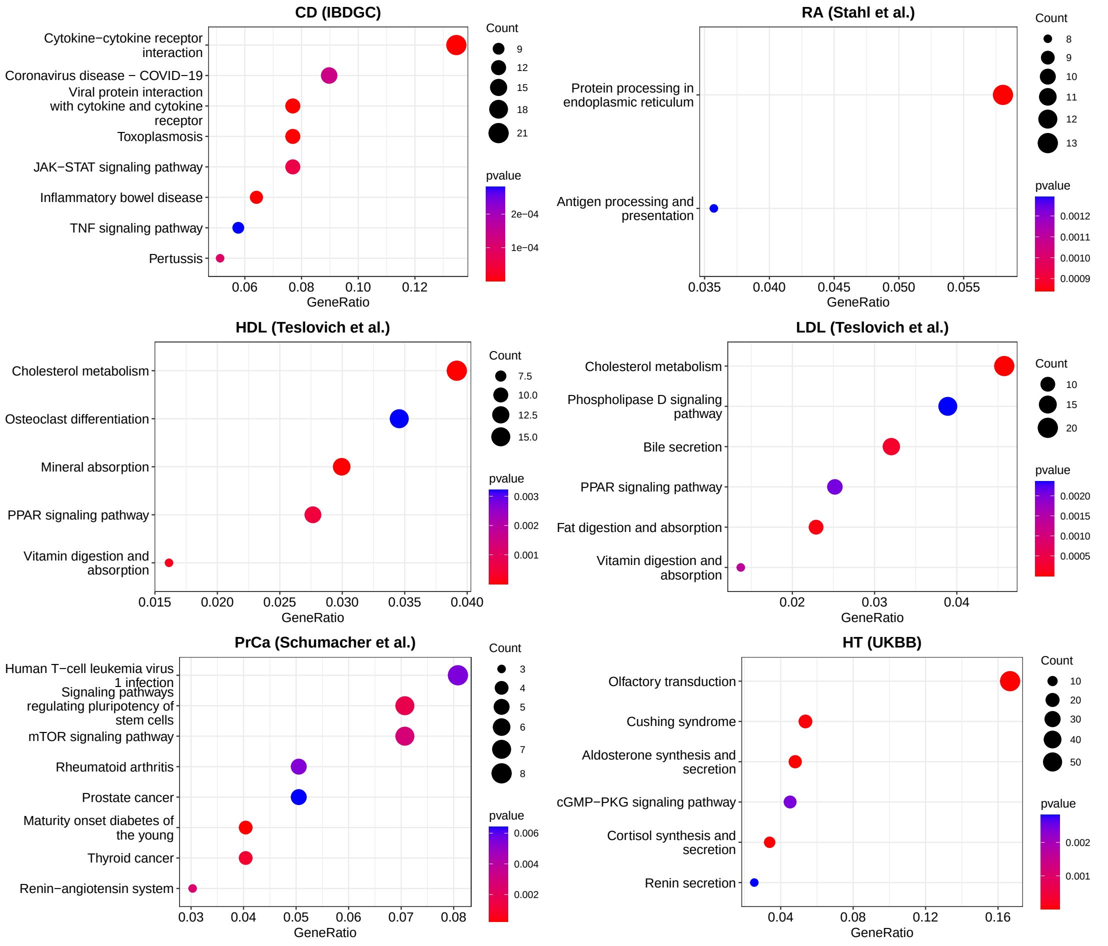

Figure S2: KEGG pathway enrichment analysis results of OWAS-joint genes (OWAS-joint $p < 5 \times 10^{-8}$). Dot plot of the enrichment analysis. The enrichment scores and gene ratio are depicted as the color and the horizon distance between the dots and y axis. The gene counts were encoded as dot sizes.

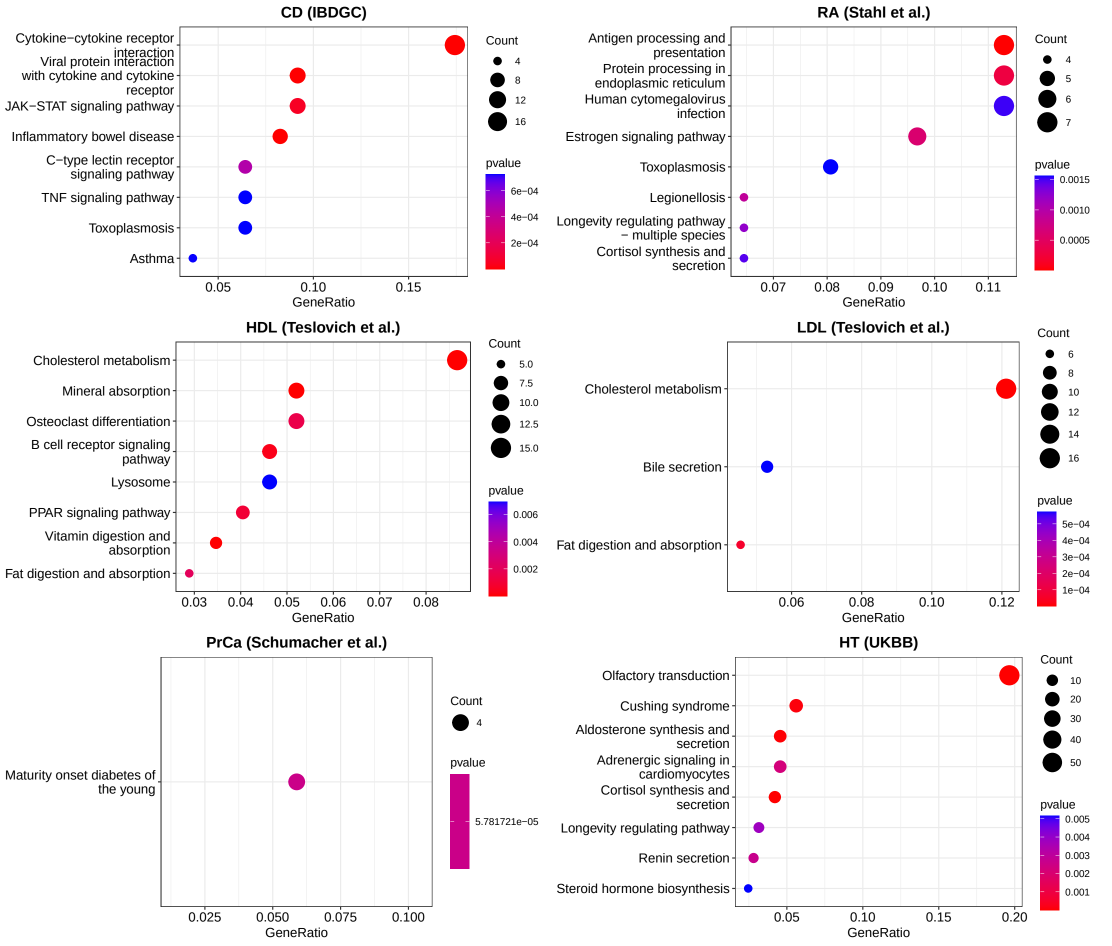

Figure S3: KEGG pathway enrichment analysis results of single-cell-type OWAS genes (OWAS $p < 5 \times 10^{-8}$). Dot plot of the enrichment analysis. The enrichment scores and gene ratio are depicted as the color and the horizon distance between the dots and y axis. The gene counts were encoded as dot sizes.
